# Microenvironmental influences on T cell immunity in cancer and inflammation

**DOI:** 10.1038/s41423-021-00833-2

**Published:** 2022-01-17

**Authors:** Darren R. Heintzman, Emilie L. Fisher, Jeffrey C. Rathmell

**Affiliations:** 1 Department of Pathology, Microbiology, and Immunology, Vanderbilt University Medical Center, Nashville, TN 37205 USA; 2 Vanderbilt Center for Immunobiology, Vanderbilt University Medical Center, Nashville, TN 37205 USA

**Keywords:** immunometabolism, T cell, microenvironment, cancer, inflammation, T cells, Translational immunology

## Abstract

T cell metabolism is dynamic and highly regulated. While the intrinsic metabolic programs of T cell subsets are integral to their distinct differentiation and functional patterns, the ability of cells to acquire nutrients and cope with hostile microenvironments can limit these pathways. T cells must function in a wide variety of tissue settings, and how T cells interpret these signals to maintain an appropriate metabolic program for their demands or if metabolic mechanisms of immune suppression restrain immunity is an area of growing importance. Both in inflamed and cancer tissues, a wide range of changes in physical conditions and nutrient availability are now acknowledged to shape immunity. These include fever and increased temperatures, depletion of critical micro and macro-nutrients, and accumulation of inhibitory waste products. Here we review several of these factors and how the tissue microenvironment both shapes and constrains immunity.

## Introduction

The classic metabolic chart present in every biology classroom and textbook is a staple of biochemical training and represents the culmination of over a century of detailed and rigorous studies. These seminal discoveries define the landscape of fundamental chemical reactions that integrate environmental signals and nutrients to support the viability, growth, and activities of every living cell. When cells receive signals to perform specific functions such as to grow and proliferate, they adjust their nutrient uptake and shift metabolic programs to meet the new demands. If, however, adequate levels of essential nutrients are not available or if end products or waste products accumulate, basic chemistry and chemical equilibriums may bring these pathways to a halt or shift their outcomes to disrupt or alter cell fates. Thermodynamic effects such as temperature changes may also influence T cells. Much the same as cell cycle checkpoints can stop or delay cell division, metabolic checkpoints thus interpret nutrients and cell energetics to determine cell differentiation, function, and fate. Cell metabolism is thus the biochemistry of how cells interpret and integrate signals from their microenvironment.

T cells and macrophage metabolism have been studied to the greatest detail in the field of immunometabolism. In addition to many studies focused on how cell activation signals can reprogram metabolism from catabolic programs designed to generate energy to anabolic programs that efficiently provide biosynthetic precursors to support cell growth and proliferation [1], it is now apparent that each immune cell type and subset has specific metabolic requirements for activation and differentiation that reflect their specific roles and demands. Importantly, the tissue microenvironment exerts a profound influence on these processes. Cell activating signals, microenvironmental nutrients, and other conditions integrate through upregulation of nutrient transporters and the subsequent changes in intracellular nutrients influence cell bioenergetics, biosynthesis, and signaling. Metabolic signaling through generation of co-factors or posttranslational modifications can then serve to shape cell fate or activate metabolic checkpoints. These include nutrient sensing pathways such as the AMPK or mTORC1 and HIF signaling axis, stress response pathways including reactive oxygen species (ROS) and ER stress, or changes to epigenetic marks and histone modifications that regulate gene expression. These changes are broadly relevant to immunity and inflammation and may have a particularly important impact in settings such as obesity or in tumor microenvironments (TME). Here we review how key changes in systemic nutrient status as well as microenvironmental metabolites and other conditions are integrated to shape the fate of T cells.

### Micronutrients and ions

**Fig. 1:**
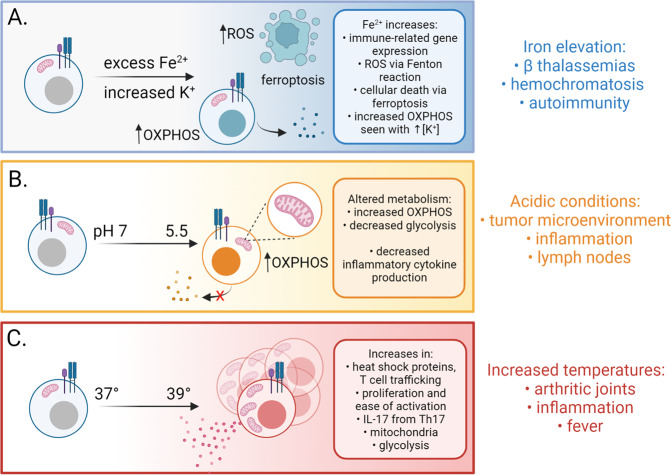
Chemical and physical components of the tissue microenvironment that can modulate immunity. **A** Changes to micronutrients, including iron and potassium, regulate T cell function and survival from ferroptosis. **B** Increased H + and decreased pH also play important roles in T cell metabolism and fate. **C** Fever and high temperatures directly impact T cells to promote inflammatory states

While cell metabolism typically focuses on intermediary metabolites and central carbon metabolism pathways, ions and other elements play key roles (Fig. 1A). Micronutrients such as ions have increasingly been shown to impact the adaptive immune system, particularly T cells. For example, recent literature has highlighted that potassium (K^+^) can directly influence T cells. Increased K^+^ ion concentration in the tumor microenvironment can acutely silence T cell effector function [2]. In contrast, high concentrations of this ion preserve T cell stemness through acetyl CoA metabolism and by epigenetically regulating gene transcription, nutrient processing, and metabolism [3]. K^+^ promoted induction of mitochondrial AcCoA synthetase 1, promoting mitochondrial metabolism, a metabolic feature of memory T cells. While these studies focused on K^+^ in the tumor microenvironment, ionic concentrations not limited to potassium have been shown to produce interesting cellular phenotypes in T cells in various tissue settings. Therefore, the ionic composition of tissues represents a relatively unexplored determinant of T cell polarization and T cell effector functions.

H^+^ concentration is a measure of acidity in tissues and high concentrations of H^+^ ions may be due to hypoxic conditions and an increased abundance of cells utilizing aerobic glycolysis and secreting lactate [4]. Most studies on acidity and T cell function have been focused on the TME and how increased lactate, and presumably acidic conditions, in the extracellular environment may inhibit effector T cell functions in solid tumors. A major consequence of low acidity seems to be through negative effects on effector cytokine production by T cells, which can be severely reduced in acidic conditions [5–7]. Acidity is, however, not only encountered in the TME and inflammatory tissues can experience pH conditions as low as ~5.5 pH [8, 9] (Fig. 1B). Interestingly, a recently published study shows evidence that lymph nodes also harbor highly acidic pH environments that suppress inflammatory effector T cell functions in T cell rich zones. In this study, naïve T cell priming was unaffected, and T cells were able to rapidly recover from acid induced inhibition within hours after pH rescue [7] such as would occur upon exit from the lymph node. This provided evidence that acidic inhibition of effector functions is related to changes in metabolic programming, as low pH shifted T cells from glycolytic metabolism to oxidative phosphorylation and mitochondrial metabolism. Future studies involving therapeutic shifts in tissue pH in inflammation and disease may aid in relieving chronic inflammation and other T cell mediated diseases.

Sodium ions (Na^+^) also influence T cell differentiation and function. Peripheral tissues accumulate NaCl in high concentrations in both diet-intake dependent, such as in high-salt western style diets, and independent mechanisms [10]. While sodium is a vital nutrient, excess salt is associated with higher incidence of autoimmune diseases such as arthritis, multiple sclerosis, and in mouse studies, colitis [11–13]. One explanation for the increased incidence of disease associated with high salt intake seems to be a shift in T cell differentiation toward the Th17 subset [12, 14–16]. Th17 differentiation in high salt conditions occurs by influencing transcriptional networks during differentiation. NFAT5 and its downstream kinase SGK1 seem to be critical for an anti-inflammatory switch in Th17 cells [17, 18]. In contrast, high salt has a more nuanced effect on regulatory T cells subsets. Studies have suggested that while induced CD4( + ) Foxp3(+) regulatory T cells are not affected in development or function by high salt [19], thymically derived regulatory T cells can become less suppressive [20]. Recently, NaCl has also been suggested to be an ionic checkpoint for human Th2 differentiation [21], further supporting a diverse role in the differentiation and function of T cells. While NaCl has not been well studied in terms of its effect on T cell metabolic programs, one may speculate that metabolic pathways are impacted in T cells by high salt. This is highlighted by the similarities between SGK1 and its close relative, Akt, a key driver of the mTORC1 pathway and T cell metabolism [22], and the shared activation of these kinases by mTORC2 [23]. These findings, as well as future studies involving functional and metabolic consequences of NaCl, have implications for a wide range of chronic inflammatory and autoimmune diseases.

Iron (Fe^+^) has received much attention in recent T cell literature. Iron deposition is a hallmark of many autoimmune diseases including lupus and multiple sclerosis and plays a key role as a co-factor for many enzymes, including TCA cycle enzymes that drive mitochondrial metabolism. Excess iron, however, can also lead to generation of ROS through Fenton reactions and lead to the process of ferroptosis, or iron-dependent cell death. Iron uptake and handling, therefore, must be tightly regulated, and aberrant iron metabolism and homeostasis have been recently shown to directly influence T cell effector functions. A direct indication of the role of iron is shown by genetic variants of the iron uptake protein Transferrin Receptor, (CD71), which are associated with a common variable immune deficiency [24]. CD71 is also a highly dynamic marker of lymphocyte activation and plasmablasts. Iron deprivation can reduce clinical scores in EAE, a T cell dependent disease in mice [25]. Conversely, excess intracellular available iron in CD4 T cells was linked to the pathophysiology of SLE through a mechanism of altered DNA methylation states favoring enhanced immune-related gene expression [26] and by regulating the stability of the RNA binding protein Pcbp1 [27]. Ferroptosis can play a key role to shape T cell responses. This process is redox mediated and largely identified through the functions of Glutathione peroxidase 4 (Gpx4), a selenoenzyme that reduces membrane phospholipid hydroperoxides to maintain cellular redox homeostasis [28]. Inactivation or depletion of Gpx4 in a variety of cell types can induce ferroptosis [29, 30]. A recent study has shown that Tregs require Gpx4 to neutralize lipid peroxides and prevent ferroptosis to maintain Treg cell activation and inhibit anti-tumor immunity [31]. In follicular helper T cells (Tfh), inhibition of ferroptosis by GPX4 also protected cells from cell death in germinal centers [32]. In tumors, CD36-mediated lipid uptake can enhance ferroptosis to dampen anti-tumor immunity by promoting lipid peroxidation [33].

### Temperature

A physical feature of tissue microenvironments that can affect thermodynamic regulation of enzymatic rates and cell physiology is temperature (Fig. 1C). Temperatures fluctuate extensively in the human body, hovering around 37 °C in the core and central organs, such as the spleen, to as low as 28 °C in peripheral organs such as the skin at thermoneutrality [34, 35]. Temperatures also vary widely in response to several physiological and pathophysiological mechanisms. Systemically, fevers can arise in many diseases and are a common side effect in bacterial and fungal infections, blood borne cancers like lymphoma, and a variety of autoimmune and inflammatory diseases, such as Adult-Onset Stills Disease [36]. While fevers are responsible for systemically increased temperatures, localized temperature changes are also common in damaged and inflamed tissue regions and have long been recognized. As early as the first century B.C., observations were made by Celsius that heat is a cardinal signs of inflammation [37]. Over 100 years ago, studies showed that after breaking the femur of a hamster, local temperatures at the break site rose by as many as 1–4 °C compared to non-injured limbs [38]. Interestingly, when ischemia was induced at the site of the break, the local temperature rose substantially, arguing that cellular activity at the site of inflammation was responsible for increased temperature and blood perfusion was necessary to dissipate this heat. These studies have been supported by more modern technological methodologies such as the use of temperature sensitive ratiometric dyes [39]. Human biology reflects these findings from mice and guinea pigs and is substantiated by several publications involving rheumatoid arthritis joints. Even in remission, rheumatoid arthritis patients can exhibit elevated temperatures in previously affected joints compared to healthy controls. In fact, the degree of temperature elevation in RA joints has been shown to be a reliable predictive indicator of disease progression [40, 41].

To date, the actual cause of locally increased temperatures at sites of inflammation is not well understood. Interestingly, mitochondrial metabolism has been suggested to generate large amounts of heat through ATP hydrolysis, with mitochondria reaching temperatures close to 50 °C within cells [42]. Notably, UCP1 expression seems to correlate with mitochondrial heat generation and is highly expressed in brown fat where non-shivering thermogenesis regulates body temperature [43]. One could speculate that mitochondrial metabolism conducted by immune cell infiltrate in inflammation may be responsible for locally increased temperatures, however this has not been tested. All together, these data suggest that heat generation and thermal characteristics within the tissue microenvironment are highly diverse, and likely important during immune challenge.

Even with a wealth of data suggestive of frequent and variable temperature change in the tissue microenvironment, the effects of temperature on immune cell function have received relatively modest attention (Fig. 1C). Elevated temperatures have been shown to promote T lymphocyte trafficking during infection through the increased expression of alpha4 integrins and Heat Shock Protein 90 [44]. Other temperature-inducible proteins like heat shock factor-1 (HSF1) are induced at a lower temperature in T lymphocytes than B lymphocytes (39 °C vs. 42 °C), indicating specialized functions of T cells at febrile temperatures [45, 46]. Several studies suggest temperature changes have significant impacts on T cell activation, proliferation, and differentiation. In vitro studies suggest that T cell proliferation occurs more rapidly and to a greater extent at febrile temperatures [47]. Elevated temperatures have also been shown to reduce costimulatory thresholds to T cell activation due to a more fluid lipid bilayer, perhaps providing a mechanism to explain elevated proliferation rates [48]. Recent work has shown that T cell differentiation is also influenced by changes in temperature. In a report where naïve CD4 T cells were primed in vitro at moderate fever temperatures (39 °C), cells underwent transcriptional reprogramming which enhanced commitment toward a Th2 phenotype and away from an IFNy producing Th1 phenotype [49]. Interestingly, coculture with dendritic cells inhibited this transcriptional transition, suggesting that cellular composition of the tissue microenvironment can influence T cell responses at febrile temperatures. Cellular signaling mechanisms have been shown to be altered by changing temperatures in other cell types, such as recent work showing that NF-kB signaling is exquisitely temperature dependent in mouse adult fibroblasts and human neuroblastoma cells [50]. An intriguing paper was recently published focusing on the effects of febrile temperatures on Th17 cell differentiation [51]. These studies provided evidence that elevated temperatures predominantly impact Th17 cell differentiation, causing this helper subset to enhance IL17a production. Febrile temperatures enhanced the pathogenic gene transcription signatures of Th17 cells and caused higher neutrophil invasion in bronchoalveolar lavage fluid in a mouse model of allergic airway inflammation. This study highlighted the potential that not only are T cells able to respond biochemically to elevated temperatures, but that this biochemical response could have T cell-subset specific effects on immunity and inflammation.

A potential explanation for subset specific adaptation to elevated temperature may be metabolic programming and mitochondrial adaptation. Exposure of CD8( + ) T cells to febrile temperatures during activation caused significant enhancement of mitochondrial respiration in addition to enhanced extracellular acidification rates [52]. RNA sequencing (RNA-Seq) of CD8( + ) cells exposed to febrile temperatures revealed that many upregulated gene pathways involved mitochondrial processes. Enzyme activity increases dramatically as temperature increases, and may predict increased metabolic rates in T cells at febrile temperatures due to faster enzymatic reactions [53]. It has been established that Th17 cells can utilize glycolysis at a much higher rate than other T cell subsets[54, 55] and seem to be especially sensitive to temperature change [51, 55]. Perhaps enzymatic activity involved in glycolysis is enhanced at febrile temperatures. This remains a poorly understood yet fundamental feature of inflammation.

### Obesity

**Fig. 2:**
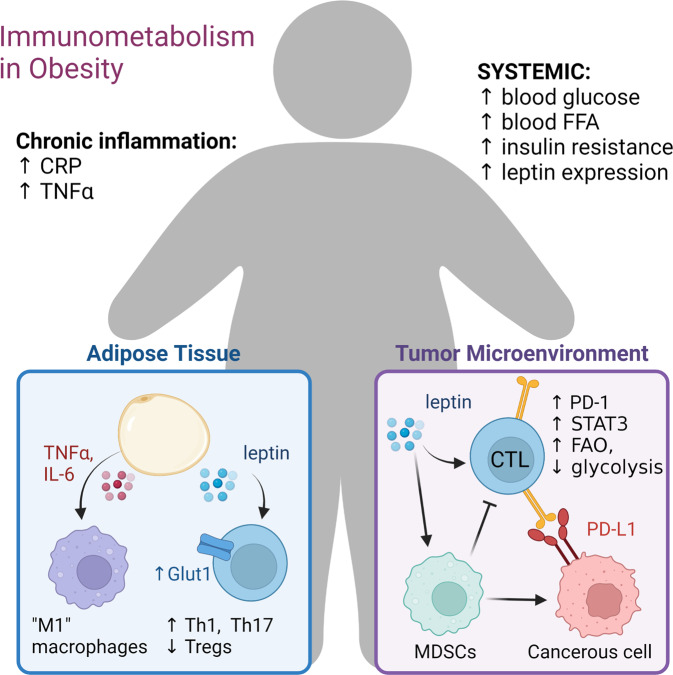
Obesity leads to both systemic and local changes to T cell microenvironments. Obesity or weight loss can have striking effects on T cell metabolism in the obesity paradox in which tumors are promoted yet sensitized to anti-PD1

Presently over 671 million adults are classified as obese (BMI > 30 kg/m2) and obesity contributing to a wide range of diseases with underlying inflammatory components (Fig. 2), including diabetes, cardiovascular disease, and cancer [56, 57]. Research on obesity has widely examined systemic effects of insulin resistance, but adipose tissue of obese individuals was also found to directly produce high levels of pro-inflammatory cytokines which influence T cell and macrophage differentiation and pro-inflammatory phenotypes, including leptin, TNFα, and IL-6 [58–61]. Cell type composition in obese tissues can also be dysregulated to favor inflammation. Obesity-associated chronic inflammation is associated with an accumulation classically activated “M1” polarized macrophages (ATMs) in adipose tissues [62, 63], which are highly inflammatory and secrete pro-inflammatory cytokines like TNFα [63]. These cells contribute to a potentially hostile environment which can shift the balance of immune cells toward a pro-inflammatory phenotype. Trem2(+) Lipid Associated Macrophages [64] are also implicated and associated with adipocyte hypertrophy, inflammation, and systemic metabolic dysregulation. While macrophages are prominent inflammatory sources in adipose tissue, mediators of macrophage and T cell polarization in adipose tissue can seemingly promote either pro- or anti-inflammatory phenotypes and include adipokines, fatty acids, and cytokines in the tissue microenvironment. The adipokine leptin, which is secreted in proportion to adipocyte mass [65], upregulates the expression of Glut1 in T cells to promote increased glucose uptake and glycolysis in T cells, fueling the expansion of T effector cells like Th1 and Th17 subsets [66]. Anti-inflammatory adipokines like Adiponectin are also present in obese adipose tissue and have been shown to limit these effector cell populations by restricting cell intrinsic glycolysis [67]. A constant battle thus maintains homeostasis within obese tissues, and dysregulation can cause meaningful swings in the outcomes of T cell differentiation and function.

A key determinant of pro- or anti-inflammatory adipose microenvironments is the frequency of regulatory T cells. Obese adipose tissue seems to become a progressively hostile environment for regulatory T cells, as they are highly present in lean adipose, but numbers decrease in obese adipose tissue [68]. Other studies have suggested that circulating regulatory T cells are also reduced in obesity [69]. One explanation for this is that regulatory T cells can be negatively influenced by adipokines like leptin, which cause regulatory T cell numbers to be reduced due to the upregulation of glycolysis [66, 70, 71]. However, dietary lipids have been shown to alter Treg cell metabolism and migratory function, promoting an effector-like migratory phenotype in Tregs, and biased migration toward sites of inflammation through a mechanism of decreased mTORC1 signaling and increased fatty acid oxidation [72]. Recently, Tregs were shown to be critical to adipose tissue homeostasis, inhibiting white adipose tissue beiging through secretion of IL-10 regulated by Blimp1 expression [73]. Interestingly, Treg-specific loss of IL-10 resulted in increased insulin sensitivity and reduced obesity in high-fat diet-fed male mice, suggesting that Tregs may promote healthy obesity in some settings. Together, these studies suggest that regulatory T cells play a unique yet currently poorly understood role in obesity.

The immunological consequences of obesity have been well studied in terms of response to viral infections. In the recent COVID-19 pandemic, obese individuals have been noted to be hospitalized with COVID-19 at a much higher rate than lean individuals [74]. Similarly, obesity has been shown to impair the adaptive immune system in response to Influenza virus [75]. Recent work has shown that T cells within obese tissue become easily exhausted through upregulation of PD-1 [76], and that PD-1 blockade can reverses T cell priming impairments seen in obesity [77]. While reversing PD-1 mediated exhaustion can be beneficial in terms of rescuing the adaptive response to viral infections, this could also increase the risk of autoimmune disorders in obese individuals. Many of the inhibitory responses in T cells due to obesity can be linked to T cell metabolism. Elevated saturated fatty acids increase T cell antigen responses and signaling through the PI3K/Akt axis to fuel fatty acid oxidation in metabolically stressed environments [78, 79]. Obesity can also result in increased T cell oxygen consumption and less effective response to pathogens, and that loss of weight and return to lean state does not rescue this metabolic change in T cells [80]. Activated CD4 + T cells from obese mice had increased glucose uptake and oxygen consumption rate (OCR), compared to T cells from lean controls, indicating increased mitochondrial oxidation of glucose [81]. Interestingly, treatment of obese mice with metformin improved T cell responses to influenza and led to increased rates of survival. Weight loss did not, however, rescue the defects in influenza response. Why weight loss does not result in the rescue of T cell dysfunction in obesity is not well understood, but epigenetic factors associated with obesity may alter T cell chromatin dynamics, creating a seemingly irreversible metabolic shift toward higher mitochondrial respiration and worse outcomes to influenza infections. Future work in this area will be of keen interest and may identify mechanisms involved in metabolic reprogramming of T cells in obesity.

### Obesity, cancer, and the immunotherapy obesity paradox

In addition to important considerations of diabetes and heart disease, it is becoming increasingly clear that obesity is associated with cancer incidence and mortality. In this section we will focus on how these factors alter the local TME (Fig. 2). The pro-tumorigenic mechanisms behind obesity are multifactorial and include direct effects of systemic hormones and nutrients on the cancer cells [82]. Chronic inflammation, long-chain fatty acid metabolism, and consumption of high fructose corn syrup have all been implicated to directly promote tumorigenesis, independent of their alterations to the anti-tumor immune response [83–85]. This adds complexity in elucidating the mechanisms involved in altering the tumor vs immune system balance and may help explain differences observed by groups in the field. Single cell RNA-Seq studies of changes to immune cell populations in tumors of lean and obese mice have shown a variety of potentially impactful changes [86, 87]. Ringel et al. showed that obesity led to increased tumor growth that was particularly apparent in immunocompetent mice and that tumors from obese mice had decreased abundance of tumor infiltrating activated CD8 T cells. They showed this was not correlated with elevated fatty acid oxidation of the T cells, but rather that T cells maintained a more naïve phenotype [86]. In addition, macrophage and monocyte populations were found to change in obesity and to have reduced expression of MHC-class II that may contribute to lower T cell activation in the TME [87].

An interesting feature of obesity-induced cancer is the duality of increased chronic systemic inflammation but reduced local inflammation and T cell exhaustion in the TME. This has been proposed to lead to the “obesity paradox”, in which obesity is a risk factor for cancer, yet has the surprising outcome of sensitizing to immunotherapy and improving outcomes upon immune checkpoint blockade (ICB) therapy [88]. This effect is seen in multiple human cancers [89–91] and recapitulated in animal models where tumors grow faster with obesity, but these same tumors can respond more thoroughly to PD-1 blockade therapy [92]. T cell dysfunction in tumors of obese animals was found to be in part driven by high levels of leptin that promoted PD-1 expression [92]. Similarly, leptin and PD-1 ligation both increased STAT3 signaling in cytotoxic T cells (CTLs) in the TME and resulted in an increase in CTL fatty acid oxidation and decreased glycolysis [93]. This led to pronounced CTL dysfunction as marked by a decrease in tumor infiltration, cytokine and granzyme B production, and tumor control. This study recapitulated previous findings suggesting STAT3 ablation increases granzyme B and CTL proliferation [94], as well as the observation that CTL dysfunction may be in part due to leptin/STAT3 signaling [89]. Leptin has also been shown to drive accumulation of myeloid-derived suppressor cells in the TME, which limit CTL activation and result in increased tumor burden [89, 95, 96]. In addition, obesity reprograms macrophages in the TME to become pro-tumorigenic [97]. Conversely, leptin can have directly pro-inflammatory roles in obesity and may sensitize macrophages with increased potential to drive inflammation [98]. Consistent with a pro-inflammatory role for leptin, treatment of lean mice with leptin was sufficient to increase anti-tumor immunity to an extent similar to PD-1 blockade [87]. Clearly, obesity and hormones such as leptin play complex roles in the TME. Given the prevalence of obesity and the potential for new insight from obesity that may improve immunotherapy in lean individuals, however, makes this an exciting and important area for further discovery.

### The tumor microenvironment

**Fig. 3:**
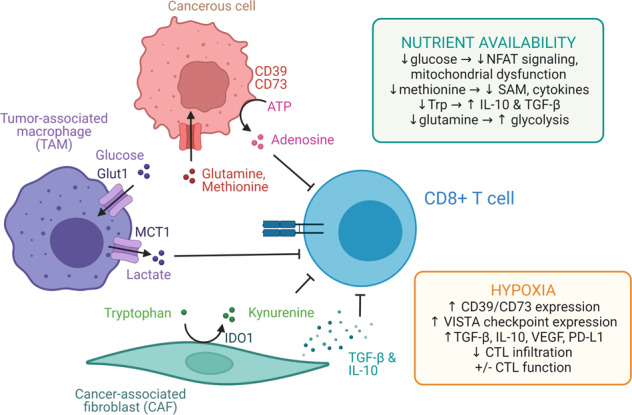
The tumor microenvironment and metabolic immune suppression. Obesity leads to chronic systemic and local inflammation. Adipose-resident CD8 T cells can be more naïve but are also sensitized to reactivate with PD-1 blockade. Many factors contribute to this obesity paradox, including elevated lactate, decreased glucose, and altered amino acids

TMEs are heterogeneous and composed of mixed cell types, nutrients, and stroma that can present a metabolically hostile setting for immunity through a metabolic immune suppression (Fig. 3). Dysplasia caused by cancer cell growth and subsequent tissue responses including recruitment of fibroblasts and immune cells disrupts normal vascular function to restrain nutrient exchange and replenishment. This is driven in part by altered metabolism caused by oncogenic signaling in the cancer cells themselves [99], although the metabolic demands of inflammation and immune cells also contribute. The complexity and heterogeneity of the TME is just beginning to be understood and several key components contribute to the ability to mount anti-tumor immune responses.

#### Hypoxia

T cell stimulation leads to activation that results in a broad increase in anabolic metabolism through aerobic glycolysis, TCA cycle metabolism, oxidative phosphorylation, the pentose phosphate pathway, and others [100]. This requires increased nutrient uptake, including oxygen to support mitochondrial metabolism. Oxygen is among the best understood nutrient sensing pathways and hypoxia is a hallmark of large or rapidly growing solid tumors. This occurs due to insufficient vascular exchange or ineffective angiogenesis and vascular maturation that cannot match oxygen consumption within the tumor. Tumor spaces often have regions with hypoxic oxygen tension well below 2% in areas distant to mature blood vessels whereas oxygen levels are near 5% in healthy tissues or adjacent to vessels. Cellular responses to low oxygen tension include induction of hypoxia-inducible factors (HIFs), most notably HIF-1α and HIF-2α, that lead to transcription of hypoxia response genes and a hypoxic stress response to increase glycolysis and anerobic metabolism [101]. In T cells, HIF-1α is also activated independent of oxygen sensing in response to TCR stimulation via the PI3K/mTOR pathway, TGF-B signaling, and IL-6 [102].

Hypoxia promotes an immunosuppressive environment through multiple mechanisms. Hypoxia induces expression of the ecto-nucleotidases CD39 and CD73, increasing TME levels of the immunosuppressive nucleotide adenosine [102]. Increased expression of immune checkpoint molecules, including V-domain immunoglobulin suppressor of T cell activation (VISTA) and cancer-associated fibroblast expression/secretion of TGF-B, IL10, VEGF, and PD-L1 also occur under hypoxic conditions [103, 104]. Consistent with an immune suppressive role for a tumor hypoxic response, targeting HIF-1α on tumor cells increased CTL infiltration and improved combination immunotherapy outcomes in a preclinical mouse melanoma model [105]. Hypoxia-targeted treatment also improved CTL infiltration and tumor control in the “immune cold” prostate cancer treated with ICB [106].

The actual role of hypoxia on CTLs, however, is complex and prior adaptation to hypoxic conditions can also increase T cell function in tumors. Increased HIF activity has been shown to increase CTL invasion and function, synergizing with ICB [107] and culturing CTLs under hypoxic conditions increased their cytotoxicity in an adoptive cell transfer model [108]. Further supporting a hypoxic response to intrinsically promote T cell anti-tumor responses, T cells made genetically deficient in VHL or the PHD proteins that lead to proteolytic degradation of HIF-1α had increased anti-tumor activity [107, 109]. A potential explanation for these different observations involves the other signals received by CTLs in the TME vs cultured in hypoxia conditions in vitro. Continuous TCR stimulation in the presence of hypoxia was recently shown to cause a Blimp-1 mediated repression of PGC-1α mitochondrial reprogramming, eventually leading to an increase in mitochondrial ROS and dysfunctional CTLs [110]. Hypoxia, therefore, may be insufficient to cause dysfunction in isolation, but remains an important factor in the complex TME.

#### Glucose

Glucose is the most abundant and prototypical carbohydrate fuel, but availability of this nutrient can be heterogeneous in the TME. Because cancer cells themselves can use glucose at high rates and vascular exchange can be poor, the overall availability of glucose in the interstitial space may be limited for cells to uptake in the TME. While glucose remains only modestly reduced and is generally available in many settings [111–113], glucose levels in some tumor regions may be as low as 0.1 mM [114, 115]. This heterogeneity may reflect regions with efficient vascular exchange relative and other regions with necrosis and high levels of death caused by insufficient nutrient access. Effector T cells, but interestingly not Treg, require high concentrations of glucose and efficient glucose uptake to elicit inflammation. Genetic deletion of the glucose transporter *Slc2a1* (Glut1) can prevent a variety of in vivo inflammatory conditions [116, 117]. Indeed, glucose restriction in CTLs leads to decreased levels of the glycolytic intermediate phosphoenolpyruvate (PEP) and decreased Ca2+ activation of the nuclear factor for T cell activation (NFAT) signaling pathway to impair cytokine production [118, 119]. Mitochondrial dysfunction, noted by small, fragmented, hyperpolarized mitochondria with decreased mitochondrial superoxide dismutase 2 (SOD2) and increased ROS, also occurs with glucose restriction [112]. Highlighting the importance of these metabolic disturbances, overexpressing PEP carboxykinase 1 to increase intracellular PEP levels, disrupting the PD-1/PD-L1 and CTLA-4 signaling pathways, and supplementation with pyruvate can increase CTL anti-tumor effector function, cytokine production, and mitochondrial ROS neutralization, respectively [112, 118, 119]. Conversely, there may be some benefit to reduced glucose uptake for anti-tumor responses. T cells that fail to differentiate to terminal effectors, may instead favor memory and long-lived states. This is potentially helpful in adoptive cell therapy or to select T cells to function in low glucose environments. Consistent with this opportunity, activation of T cells in the glycolytic inhibitor 2-deoxyglucose (2DG) delayed T cell activation and effector function to shift nutrient use and allow greater in vivo viability and persistence that increased anti-tumor immunity [120, 121]. Conversely, Treg do not require high levels of glucose and can function without Glut1 [116].

While assays to measure glucose uptake and accessibility in tumors suggested models where glucose limitation restricts anti-tumor immunity, these studies have typically been performed with bulk tumor tissue, with non-specific dye indicators [122], or at low levels of resolution that preclude insight to which cells can capture available glucose in the TME. This has led to a nutrient competition model in which cancer cells consume the bulk of the available glucose and cause a metabolic immune suppression by limiting T cell access to this important nutrient [114, 123]. To directly test this model, radiolabeled Positron Emission Tomography tracers were given to tumor bearing mice and tumor infiltrating cells were fractionated to determine which population internalized the labeled nutrient [113]. Interestingly, ^18^F-2DG was primarily taken up by macrophages, followed by T cells and cancer cells while ^18^F-glutamine was primarily taken up by cancer cells followed by macrophages and T cells. Treatment with a glutamine uptake inhibitor, however, increased glucose uptake in all cell populations. These data show that in most settings, glucose is broadly available in the TME and that macrophages rather than cancer cells are the dominant glucose consumers. Importantly, the ability of cells to increase glucose uptake upon inhibition of glutamine uptake demonstrated that glucose uptake was not widely limited by accessibility, but rather by cell intrinsic metabolic and signaling programs.

#### Amino acids

Growing evidence has highlighted the importance of amino acid to modulate anti-tumor responses. A wide range of amino acids including arginine, and serine or glycine have been shown to be critical for anti-tumor effector T cell function [124] and T cell activation even in glucose replete conditions [125]. As the most abundant amino acid, glutamine is utilized as an anabolic and anaplerotic nutrient by cancerous cells and effector T cells [113, 126]. Effector T cells require glutamine uptake as genetic deletion of the transporter Asct2 or SNAT2 impair effector T cell responses [127–129]. Following uptake, glutamine is used in nucleotide or hexosamine synthesis, transported back out of cells in exchange for other amino acids, or converted to glutamate via the enzyme glutaminase (Gls). Gls generation of glutamate then supports glutathione synthesis, methylation reactions, catabolism, and the TCA cycle substrate via conversion to alpha-ketoglutarate. Despite these widespread and important roles for glutamine, genetic or pharmacologic disruption of Gls had subset-specific effects on T cells that showed that excessive glutamine uptake can be immunesuppressive [130]. Th17 cells required Gls and Th17-mediated inflammatory disorders were prevented by Gls inhibition. In contrast, Th1 cells and CTLs responded to Gls inhibition by compensating through increased glucose uptake and glycolysis that increased mTORC1 signaling, glycolysis, and production of IFNy, granzyme B, and perforin [130]. This observed phenotype of increased activation has since been explored by multiple groups, showing an increase in anti-tumor killing of CTLs treated with GLS inhibitors (telanglenastat, or CB-839, and BPTES) when coupled with checkpoint immunotherapy [131–133]. In one study, glutaminolysis inhibition resulted in a decrease in GSH, which in turn led to global reduction in glutathionylation in the cell, including SERCA glutathionylation that increased NF-kB signaling and PD-1 expression [131]. In addition to specific targeting of Gls, glutamine metabolism can be broadly inhibited using the glutamine analog 6-diazo-5-oxo-L-norleucine (DON). DON, and a modified DON molecule to target the TME more directly due to toxicity concerns, reduced tumor burden even when used as a monotherapy [134]. This approach both targets a metabolic pathway preferentially used by cancer cells [113] and promotes metabolic pathways favored by anti-tumor T cells [130, 134]. While promising, much remains to ensure T cells do not exhaust and that essential glutamine metabolic pathways are not also suppressed to ultimately limit T cell persistence or function.

Cancer cells can also be dependent on methionine, and limiting methionine may bolster current therapies [135]. Indeed, methionine restriction can lead to decreased tumor burden in immunocompromised mice to support a non-immune role for methionine in cancer growth [135, 136]. However, activated T cells upregulate and sustain methionine transporters, and methionine restriction can decrease cytokine expression and increase apoptosis [137, 138]. Specifically, methionine depletion resulted in decreased intracellular SAM concentrations, loss of demethylation at lysine 79 of histone H3 via leukemia associated methyltransferase disruptor of telomeric silencing 1-like (DOT1L), and functionally impaired CTLs [138]. Conversely, methionine supplementation, rather than restriction, may reduce tumor burden in immunocompetent systems [138]. De novo methionine synthesis also appears essential for maximal T cell proliferation in vivo, as a CRISPR screen of genes in the methionine cycle of one-carbon metabolism showed a loss of T cell fitness if Mat2a, Mtr, or Mtrr were disrupted [139]. While further research is needed to establish mechanistic details, our growing understanding of methionine in the TME highlights the importance of studying metabolic alterations in immunocompetent systems, as nutrient availability alters the survival and function of immune cells as well as cancerous ones.

Metabolism of the essential amino acid tryptophan by the enzyme indoleamine 2,3-dioxygenase 1 (IDO1) of suppressive DCs, TAMs, and CAFs results in both the depletion of tryptophan and accumulation of the immunosuppressive metabolite kynurenine. As an essential amino acid, tryptophan must be obtained through the diet, and it cannot be replaced if it becomes limiting in tissues. IDO1 is overexpressed in most cancers, and kynurenine levels in the TME correlate with poor prognosis in cancers such as melanoma, colon cancer, ovarian cancer, and AML [140–142]. Kynurenine binds to the aryl hydrocarbon receptor in naïve CD4 + T cells, promoting Treg differentiation [142]. In addition, the depletion of tryptophan in the TME activates the stress-response kinase GCN2 in T cells, which both inhibits proliferation and induces differentiation into Tregs. GCN2 in DCs and TAMs also leads to expression of inhibitory cytokines such as IL-10 and TGFB, leading to a suppressive milieu [143]. Given the role of IDO1 creating this environment, it has been the target of multiple preclinical and early clinical trials over the past few years, particularly in combination with ICB [142, 144]. While initial trial results were not positive, there remains a high potential for effective combination therapies or in specific patient subsets.

#### Metabolites that accumulate to suppress immunity

In contrast to the depletion of anabolic nutrients in the TME, many metabolites in addition to kynurenine can accumulate in local microenvironments as waste or secreted products to inhibit T cells. This is particularly the case in tumors or inflamed tissues where vascular exchange is poor. Levels of adenosine can be elevated in the TME due to the release of ATP upon cell lysis which is converted to ADP and adenosine by the ectonucleotidases CD39 and CD73 on the surface of tumor cells. Adenosine may then act on the A2A- and A2B-receptors on T cells and APCs, respectively, to reduce CD8 T cell activation, proliferation, and anti-tumor function [145]. Local accumulation of this immunosuppressive molecule impairs the effectiveness of therapeutic approaches that induce ATP release via tumor cell lysis, prompting multiple groups efforts to disrupt conversion and adenosine signaling [145, 146]. For example, utilizing a CRISPR/Cas9 approach to disrupt A2AR on CAR-T cells in culture led to an increase in IFNγ, TNFα, JAK/STAT pathway genes, CAR-T cell survival, and control of tumor burden in a murine breast cancer model [147].

As a result of elevated glucose uptake and glycolysis in tumors that lead to a demand to convert NADH back to NAD by Lactate Dehydrogenase, large quantities of pyruvate are shunted to lactic acid formation rather than entering the TCA cycle. This can lead to an interstitial accumulation of lactate and decreased pH. This acidification has been shown to reduce anti-tumor CTL activation, glycolysis, and expression of functional markers such as IFNγ via diminished NFAT signaling [148, 149]. In addition, the checkpoint molecule VISTA suppresses T cells selectively in acidic environments and is upregulated in the TME [150]. While lactate can inhibit effector T cells [151], it is not just as a waste product and can serve as a metabolic fuel and signaling molecule. Treg are oxidative and can take up and consume lactate in the TME [152, 153]. Clinically, levels of lactate dehydrogenase A (LDH-A), which converts pyruvate into lactate, correlate with worse clinical outcomes and fewer infiltrating T cells in multiple cancer types [148, 154, 155].

Lactate accumulation in the TME is a rationale target for anti-tumor preclinical research, yet there are complexities to the feasibility of improving therapies via targeting this mechanism. While LDH-A silencing in tumor cells via shRNA failed to alter lactate levels, pH, or cell survival in the TME [156], a subsequent study utilizing nanoparticle delivery of shRNA did show a reduction in lactate, pH neutralization, and increased CTL infiltration and tumor control [157]. Complicating the picture, another study found tumor specific LDH-A knockdown with shRNA enhanced CAR-T treatment and reduced tumor growth, however lactate levels and pH in the TME were unchanged [154]. LDH-A was also shown to play a role to regulate epigenetic marks through histone acetylation to promote IFN-γ expression [158]. In addition, TME neutralization has occurred in studies utilizing bicarbonate delivery and inhibition of the lactate exporter monocarboxylate transport 1 [149, 159]. One potential explanation for these discrepancies is the differential expression of LDH-A vs the LDH-B isoform in various tumors [156]. In addition, local lactate levels are dependent on both tumor cell apoptosis and regional vascularization, which vary widely between treatment conditions and tumor type. Importantly, when these lactate-targeting treatments did improve outcomes, multiple studies showed this to be a CTL-dependent effect and treatment with LDH shRNA synergized with PD-1 ICB yet failed to improve tumor outcomes in immunodeficient systems [157]. Supporting this notion, LDH inhibition controlled tumor growth in a preclinical humanized non-small cell lung cancer model in a CD8 + T cell dependent manner [160]. It was recently shown that preconditioning CTLs with LDH-A inhibition resulted in less terminally differentiated CTLs upon IL-2 treatment, while treatment with IL-21 did not alter cellular metabolism but did decrease transcription of LAG3, PD-1, and TIM3, leading to an overall increase in cell persistence, tumor control, and host survival [161].

### Conclusions and perspective

In as much as it is now apparent that metabolic pathways are intimately linked to T cell fate, it is also clear that local nutrients and physical conditions influence these processes. While we reviewed several factors here, our understanding of these processes and nutrient availability remains poor. These questions are challenged by the need to consider in vivo tissue heterogeneity and the complexity of different cell types and activation states. Nevertheless, the widespread and necessary use of biochemical and bulk tissue assays obscure cell heterogeneity in many settings. The variety of important factors for immunity in tissues is beyond the ability to accurately model or quantitate using simplified in vitro. It is important, however, to focus studies on in vivo systems and consider microenvironmental factors and tissue heterogeneity where possible. It is likewise important to consider how different cell types may interact through metabolites and how they may respond differently to the same nutrient pool. These processes may have important implications for immune cell function in different tissues, such as tumors relative to lymph nodes, gut, adipose, liver, skin, or other disparate organ sites. They also, however, offer an exciting opportunity to modulate immunity in tissue and cell type specific ways with implications for a wide variety of inflammatory conditions or cancer types.
